# Temporal dynamics of offline transcranial ultrasound stimulation

**DOI:** 10.1016/j.crneur.2025.100148

**Published:** 2025-03-06

**Authors:** Cyril Atkinson-Clement, David Howett, Mohammad Alkhawashki, James Ross, Ben Slater, Marilyn Gatica, Fabien Balezeau, Chencheng Zhang, Jerome Sallet, Chris Petkov, Marcus Kaiser

**Affiliations:** a Precision Imaging, School of Medicine, University of Nottingham, United Kingdom; b School of Psychological Science, University of Bristol, United Kingdom; c Biosciences Institute, Newcastle University Medical School, United Kingdom; d NPLab, Network Science Institute, Northeastern University London, London, United Kingdom; e Department of Neurosurgery, Ruijin Hospital, Shanghai Jiao Tong University School of Medicine, China; f Shanghai Research Center for Brain Science and Brain-Inspired Intelligence, China; g Wellcome Centre for Integrative Neuroimaging, Department of Experimental Psychology, University of Oxford, United Kingdom; h Univ Lyon, Université Lyon 1, Inserm, Stem Cell and Brain Research Institute U1208, Bron, France; i Department of Neurosurgery, University of Iowa, USA; j School of Computing Science, Newcastle University, United Kingdom; k Rui Jin Hospital, Shanghai Jiao Tong University, Shanghai, China

**Keywords:** Ultrasound neuromodulation, Seed-based connectivity, fALFF, ReHo

## Abstract

Transcranial ultrasound stimulation (TUS) is a promising non-invasive neuromodulation modality, characterized by deep-brain accuracy and the capability to induce longer-lasting effects. However, most TUS datasets are underpowered, hampering efforts to identify TUS longevity and temporal dynamics. This primate case was studied awake with over 50 fMRI datasets, with and without left anterior hippocampus TUS. We therefore amassed the highest-powered TUS dataset to date required to reveal TUS longevity and dynamics. Most of the effects were found in the TUS region itself and alongside the default mode and sensorimotor networks. Seed-based functional connectivity exhibited a time-constrained alteration which dissipated ∼60 min post-TUS. Intrinsic activity measure and regional homogeneity displayed extended diffusivity and longer durations. This high-powered dataset allowed predicting TUS using pre-stimulation features that can now extend to modeling of individuals scanned less extensively. This case report reveals the diversity of TUS temporal dynamics to help to advance long-lasting human applications.

## Highlights

• TUS “*offline*” effects on the primate brain accumulate and last many minutes.
• TUS changed functional connectivity withing a window of 60 min.
• TUS changed intrinsic activity and regional homogeneity for at least 100 min.
• TUS effects could be predicted based on the high-powered control dataset.

## Graphical abstract

**Figure:**
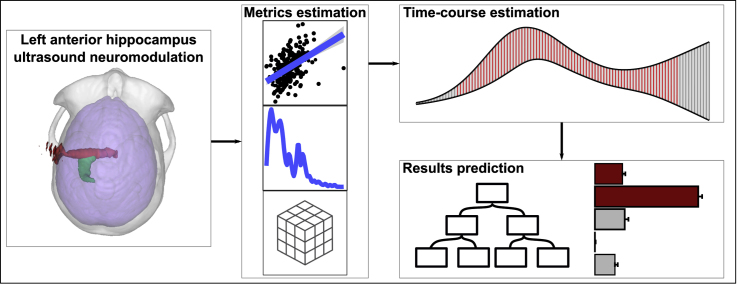

## 1 Introduction

The extrinsic manipulation of brain function holds significance for both clinical and transhumanist objectives. In the former context, the aim is to rectify dysfunctions arising from diverse neurological or psychiatric conditions, whereas in the latter, the goal is to enhance normal neural operations. Various strategies have been developed to achieve these ends, with pharmacology or surgically invasive deep brain stimulation being the primary modalities. However, these methods often lack sufficient neural target localization or are excessively invasive for human application. This has spurred the emergence of non-invasive approaches like transcranial magnetic stimulation and transcranial direct current stimulation, characterized by their non-invasive nature, reduced discomfort, and enhanced precision compared to medication. Despite their advancements, these techniques are hampered by two primary limitations: limited spatial accuracy and inability to directly target deep-seated brain regions.

Addressing the limitations of prior non-invasive methods, the recent development of low-intensity transcranial ultrasound stimulation (TUS) offers the potential to effectuate brain function changes noninvasively with high spatial precision and unmatched penetration depth (Bault et al., 2023; Blackmore et al., 2023). This innovation holds the promise of significant advances in neuroscience when combined with methods to visualize neural functions such as functional MRI. However, the nascent state of TUS neuromodulation translates into a limited understanding of its potential longevity of effects and neural impact.

Low intensity TUS involves the application of a mechanical sound pressure wave within a frequency range of 200 kHz to 10 MHz or higher, capable of inducing transient modifications in neuronal activity without inducing damage through tissue heating (Naor et al., 2016; Gaur et al., 2020). Although its precise mechanism remains a topic of debate, several non-exclusive hypotheses are prominent: TUS could induce mechanical opening of mechanosensitive voltage-gated ion channels (Kubanek et al., 2016, 2018; Tyler et al., 2008; Yoo et al., 2022); it could induce localized depolarization through sonic micro-cavitation (Krasovitski et al., 2011; Plaksin et al., 2016); or it could impact the coupling between glial cells and neurons (Oh et al., 2019).

To date, most studies exploring TUS effects have focused on immediate “*online*” stimulation, yielding insights into its immediate or short-term impact on brain functioning. Yet, a common assumption is that TUS effects can be longer-lasting and are relatively stable in temporal dynamics throughout the brain network or gradually dissipate in the neural network over a currently poorly understood time course. Based on “*offline*” studies (i.e., neuroimaging obtained after TUS), various indications suggest that TUS can induce transient effects lasting from several minutes to several hours. In primates, the application of TUS to cortical or deep cortical/subcortical regions led to alterations in brain connectivity enduring over an hour (Folloni et al., 2019; Verhagen et al., 2019). Monkeys receiving TUS targeting the anterior cingulate cortex exhibited changes in functional connectivity and cognitive performance during a counterfactual choice task more than 30 min after stimulation (Fouragnan et al., 2019). In humans with chronic pain, TUS administered to the posterior frontal cortex contralateral to the source of maximal pain elicited significant mood enhancement after 40 min (Hameroff et al., 2013). Similarly, TUS applied to the right prefrontal inferior cortex in healthy humans induced a positive mood effect up to 30 min after stimulation, accompanied by alterations in functional connectivity within the default mode network (Sanguinetti et al., 2020). Also, TUS was found to reduce GABA levels in the brain target for at least 50 min following the stimulation (Yaakub et al., 2023). Lastly, one *in vitro* study with a non-human animal model reported that offline TUS could last for up to 8–12 h (Clennell et al., 2021).

Evaluating the time course of TUS temporal dynamics has been hampered by the lack of high-powered datasets with long scanning times and a high number of scans from the same individual, amenable to neural system analytics. Such a dataset would be a tour de force in any given individual, and a high-powered case study could provide a substantial breakthrough in understanding the impact of TUS dynamics for other individuals.

The present case report allowed amassing substantial amounts of data in a single macaque monkey with and without TUS of the left anterior hippocampus for dynamical analytics and modelling. We selected the hippocampus due to its central role in cognitive function, its prominence within the brain network due to its hub position (Schultz et al., 2014; Wagner et al., 2016), its relevance to various diseases (Small et al., 2011) and its frequent indirect stimulation through other non-invasive brain stimulation (i.e., targeting another part of the brain well connected to the hippocampus to change its brain functioning) (Freedberg et al., 2019; Hermiller et al., 2018; Thakral et al., 2020; Velioglu et al., 2021; Wang et al., 2014). The primary objective was to elucidate how TUS can modulate brain-wide function and to analytically discern the time-course of these alterations across several neural measures throughout a time window up to 100 min following TUS. Based on assumptions of longer lasting effects reported in the literature with unknown longevity, the key hypothesis that we tested is of comparable TUS effects across neural measures gradually dissipating throughout the brain following TUS application. The results did not support this hypothesis, showing a differential extended time course of effects on the neural network for as long as we were able to measure (at least 100 min).

## 2 Results

### 2.1 Experimental details and analysis strategy

**Fig. 1:**
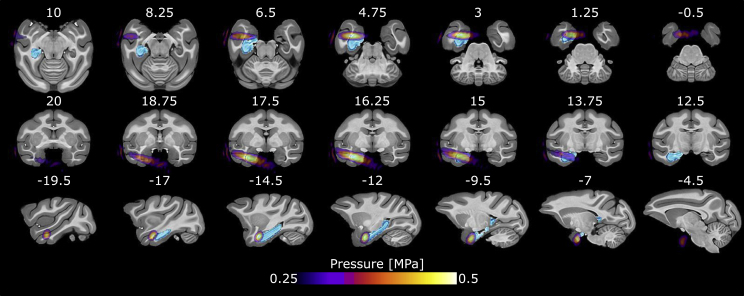
Details of the sonification simulation using k-Plan. The figure represents the applied acoustic pressure (colored) on the template space. The whole hippocampus is represented in blue.

For this study, one Rhesus macaque (*Macaca mulatta*, male, 15 years old, 13.5 kg) underwent 52 resting-state fMRI (rs-fMRI) obtained awake and distributed in 21 daily scanning sessions. 27 scans (12 sessions) were obtained without any stimulation (“control”) while 25 (9 sessions) were obtained between 32 and 91 min following TUS applied on the left anterior hippocampus (see Fig. 1; mean = 45.8min; standard deviation = 12.9min; quartile 1 = 32–46min; quartile 2 = 46–54min; quartile 3 = 54–61min; quartile 4 = 61–91min). The 32–91 min period before the scanning started was because it took several minutes after TUS or sham (placing the TUS transducer on the animal’s head but not stimulating) to continue to prepare the animal for the scanning session including preparing the imaging coils around the head, advancing the animal to the center of the bore and conducting the scanner preparation scans before commencing the fMRI data acquisition.

The TUS was applied on the left hippocampus by using a commonly used primate “offline” protocol (Folloni et al., 2021) (250 kHz, 30 ms bursts, 100 ms period duration, 30% duty cycle, total duration of 40 s), see Methods for details and TUS simulations. This acoustic simulation confirmed that the left anterior part of the hippocampus was the target of TUS (areas with an estimated applied pressure >0.2 MPa: left area TE [355 voxels], left hippocampal formation [265 voxels], left rostral superior temporal region [242 voxels], left rhinal cortex [175 voxels], left pallial amygdala [145 voxels], left fundus of the superior temporal sulcus [100 voxels], left striatum [37 voxels], left caudal superior temporal gyrus [25 voxels], right rhinal cortex [15 voxels], left belt areas of the auditory cortex [8 voxels], left subpallial amygdala [6 voxels], left core areas of the auditory cortex [1 voxel]).

**Fig. 2:**
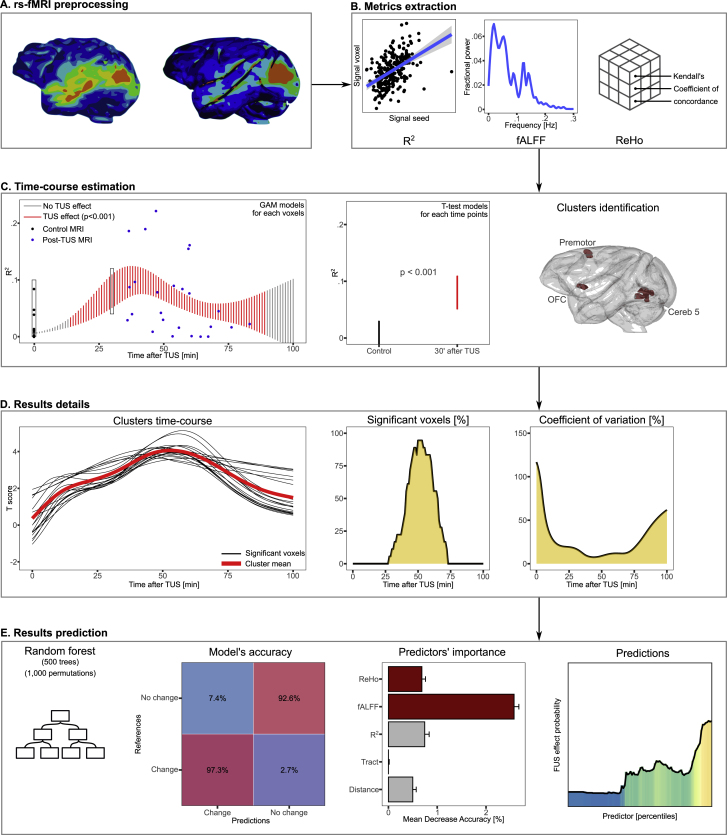
Overview of the analyses process. A. Illustration of the rs-fMRI pre-processing (slice timing correction, despike, motion correction, realignment, smoothing, denoising). B. Illustration of the 3 considered metrics (i.e., R^2^, fALFF and ReHo). C. Statistical approach to extract the time-course of the changes induced by TUS. This step consisted of one beta generalized additive model (GAM) between the time and the metric, and of t-tests for each time points between the control and the model’s estimated values. D. Illustration of the results details' estimation, including the estimation of the mean time-course of the changes induces by TUS for each significant voxels of a significant cluster, the percentage of significant voxels within the cluster and the coefficient of variation at each time point. E. Illustration of the process of the random forest model with the confusion matrix between the real and the predicted classification, the mean decrease accuracy of each predictor and the probability to observe a TUS effect according to the ranking of a significant predictor.

We used several imaging features to observe functional changes after TUS stimulation of the left hippocampus (see Fig. 2B and Methods for details): seed-based resting-state functional connectivity (R^2^; using a sphere of 2.5 mm diameter, x = −14, y = 14.25, z = 4.25), mean fractional Amplitude of Low Frequency Fluctuations (fALFF) and the mean regional homogeneity (ReHo). fALFF is a measure of spontaneous fluctuations which could be interpreted as spontaneous neural activity, with the caveat that fMRI BOLD does not directly measure neurophysiological responses (Cordes et al., 2001; Zou et al., 2008). ReHo is a measure of local functional connectivity between a voxel and its nearest neighbors which could be interpreted as local synchronization (Jiang and Zuo, 2016). ReHo is calculated using the Kendall’s coefficient of concordance (Kendall and Gibbons, 1990) to determine the degree of similarity between the time series of one voxel and those of its 27 neighbors. A high value indicates a high similarity between a voxel and its neighbors. These three metrics are among the most commonly used in fMRI studies. As they all provide different information, their combination offers a clear picture of the brain’s functioning.

On these metrics, we investigated brain changes following TUS (25 rs-fMRI scanning runs; ∼11 min duration each; taking advantage of the variability in the fMRI acquisition starting time between 32 and 91 min following TUS, the required fMRI setup time to scan the primate) over-time using a generalized additive model (GAM) to smooth the time-course of the observed changes and extrapolate our observation window from 0 to 100 min following TUS. Two-samples t-tests allowed conducting comparisons with the control measures (i.e., without TUS; 27 rs-fMRI sequences; see Fig. 2C and D).

We then used random forest model (Kam Ho, 1998) coupled with permutations to identify if control features (i.e., obtained before TUS) could be relevant to predict TUS effects (see Fig. 2E). For this, we generated a dataset with a combination of neural metrics (i.e., mean R^2^, mean fALFF, mean ReHo, as well as the Euclidean distance and the structural connectivity between the target and rest of the brain) for each grey matter voxel, excluding those within the target sphere. Each voxel was attributed to a “change”/“no change” group if it was identified as altered based on the GAM/t-tests analyses as described above. The aim of the model was to use the control features to predict the TUS stimulation/no-stimulation grouping.

To determine the extent to which the full dataset was necessary for the obtained results and if the results could be observed with a smaller amount of data from this dataset, we reran the analyses on the voxels found as significant with the entire dataset by selectively removing data from it. With this approach we were able to determine the minimal dataset size required to observe the main effects with the fully powered set (see appendix).

Last, we performed additional analyses to address questions of non-specific temporal confounds not related to the TUS conditions and whether TUS effects accumulate at least on the next TUS session. As a measure of stability, we determined if the values observed in the peak of each clusters were significantly correlated with, 1) the session order for all fMRI run which included no-TUS control sessions (which could be interpreted as a stability measure over the study, so mostly as a noise control and/or control for unspecific changes in time not directly related to TUS effects); and 2) for the TUS sessions only (which gives some insight about carry over effects; see Table 4).

### 2.2 TUS changes functional connectivity in a time-constrained manner

**Fig. 3:**
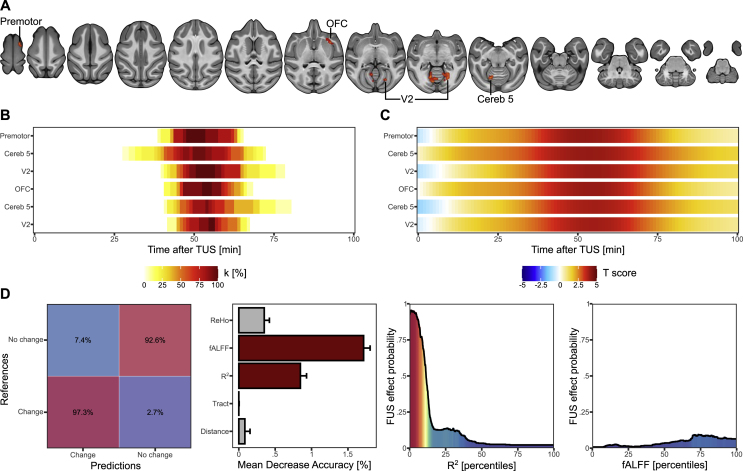
Anterior left hippocampus TUS effects on seed-based functional connectivity. A. Spatial location of the significant clusters showing an increased (red) or a decreased (blue) connectivity following TUS. B. Percentage of the clusters which are significant from 0 to 100 min after TUS. C. Mean T-score of the clusters which are significant from 0 to 100 min after TUS. D. Results of the random forest model with, from the left to the right, the confusion matrix between the real and predicted classification of each voxel, the mean decrease accuracy of each predictor (grey for non-significant predictor and red for significant predictor), the probability to observe a change of functional connectivity according to the R^2^ and the fALFF ranking.

We found that TUS alters the functional connectivity between the left anterior hippocampus and several other brain regions in a time-constrained manner. The alteration occurred between 43 and 62 min after stimulation, and it was exclusively an increase in connectivity (see Fig. 3, panel A for the localization of the significant clusters, panel B for their size over time and panel C for the effect size over time). Specifically, hippocampal connectivity was increased with the left cerebellum (k [cluster size in voxel] = 18, significant from 43 to 62 min; k = 13, significant from 49 to 59 min), the right occipital V2 cortex (k = 12, significant from 46 to 62 min; k = 10; significant from 49 to 60 min), the right caudal part of the orbitofrontal cortex (k = 12, significant from 46 to 61 min) and the right premotor cortex (k = 10, significant from 44 to 61 min; see Table 1).

**Table 1:** Details of the significant clusters for the effects of TUS on R^2^.

Clusters	Peak	Peak [x y z]	T	K	Details	Effect duration after TUS [min]
**1**	Cereb 5 (L)	−6 -9.5 13.5	5.141	18	Cereb 5 intermediate (k = 8) Cereb 6 intermediate (k = 6) Cereb 5 vermis (K = 3) Cereb simple lobule (k = 1)	43–62
**2**	Cereb 5 (L)	−4.75 -12.25 13.5	4.668	13	Cereb 5 vermis (k = 12) Cereb 6 intermediate (k = 1)	49–59
**3**	V2 (R)	11–9.75 12.25	4.549	12	V2 (k = 8) Ventral V3 (k = 2) Cereb simple lobule (k = 2)	46–62
**4**	OFC (R)	18 23 19.75	4.645	12	Claustrum (k = 7) Gustatory cortex (k = 3) Lateral OFC (k = 2)	46–61
**5**	V2 (R)	11–12.25 17.25	4.490	10	V2 (k = 5) V1 (k = 5)	49–60
**6**	Premotor (R)	4.75 20.5 38.25	4.671	10	Dorsal premotor cortex (k = 10)	44–61

The random forest model demonstrated a 92.65% (p < 0.0001) accuracy in detecting voxels with altered R^2^ due to TUS, and high percentage of true negative (voxels with no change identify as having not changed) and true positive (voxels with a change identify as having changed) identifications, respectively of 92.6% (p < 0.0001) and 97.3% (p < 0.0001; See Fig. 3D). Using a permutation approach, we gauged the importance of each predictor in achieving this overall accuracy. Among the five employed metrics, two emerged as significant: the fALFF (mean decrease accuracy = 1.71% ± 0.09%, p = 0.0009) and the R^2^ (mean decrease accuracy = 0.0.84% ± 0.08%, p = 0.009). Conversely, the ReHo, Euclidean distance and number of tracts were not significant (mean decrease accuracy <0.34%, p > 0.16). Moreover, model prediction allows us to determine the values of the two most significant predictors which are likely to be related to a TUS induced change in R^2^ after controlling for all the other metrics (by forcing their values to be on the median): a fALFF of 0.485 (percentile 73.5, 9.4% likely to obtain a changed R^2^ after TUS) and a R^2^ of 0.006 (percentile 1, 95.4% likely).

We identified that a significant amount of data is required to observe these results (see appendix). The stronger cluster corresponds to the right premotor cortex (50% of full dataset effects remained significant with 14/25 data and 75% with 16/25) followed by the left cerebellum (50% with 16/25 data). Conversely, the orbitofrontal cortex, the right occipital V2 cortex and the orbitofrontal cortex tend to completely disappear with less than 13/25 data. Four clusters were found as significantly related to the potential confound of fMRI acquisition session order across the TUS and no-TUS sessions (i.e., the left cerebellum, the two clusters in the right occipital V2 cortex, the right premotor cortex), but none of these survived after Bonferroni correction (see Table 4).

### 2.3 TUS changes intrinsic brain function in a time-diffuse manner

**Fig. 4:**
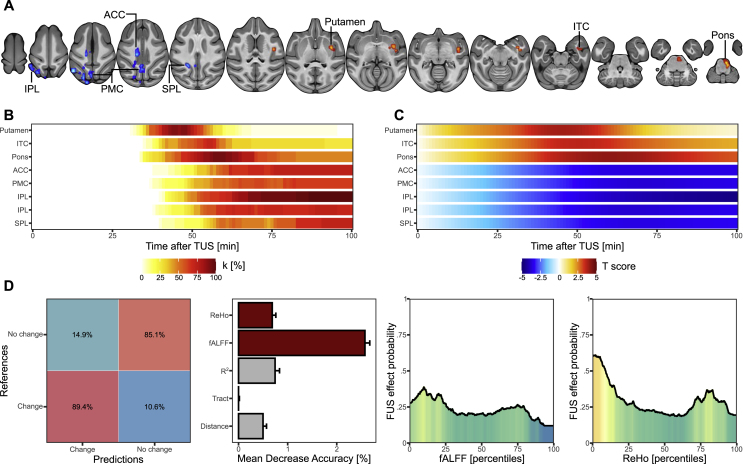
Anterior left hippocampus TUS effects on fALFF. A. Spatial location of the significant clusters showing an increased (red) or a decreased (blue) fALFF following TUS. B. Percentage of the clusters which are significant from 0 to 100 min after TUS. C. Mean T-score of the clusters which are significant from 0 to 100 min after TUS. D. Results of the random forest model with, from the left to the right, the confusion matrix between the real and predicted classification of each voxel, the mean decrease accuracy of each predictor (grey for non-significant predictor and red for significant predictor), the probability to observe a change of functional connectivity according to the fALFF and the ReHo ranking.

**Fig. 5:**
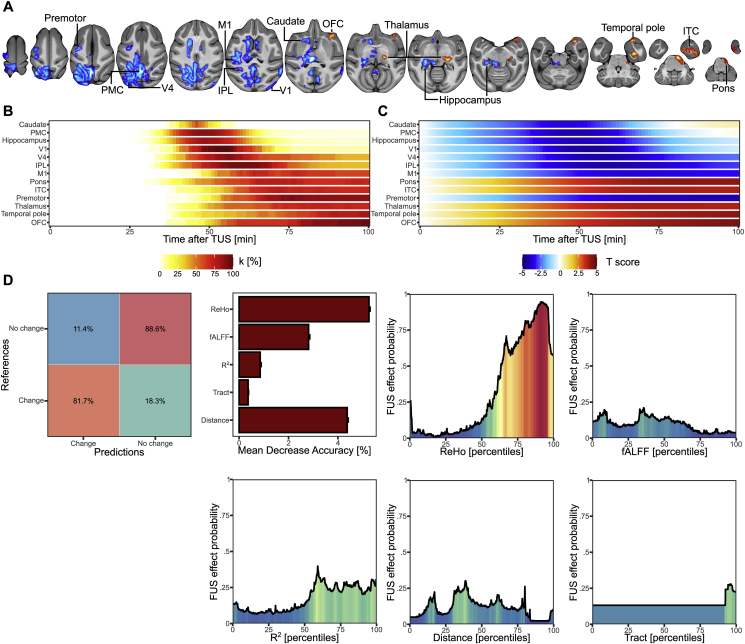
Anterior left hippocampus TUS effects on ReHo. A. Spatial location of the significant clusters showing an increased (red) or a decreased (blue) ReHo following TUS. B. Percentage of the clusters which are significant from 0 to 100 min after TUS. C. Mean T-score of the clusters which are significant from 0 to 100 min after TUS. D. Results of the random forest model with, from the left to the right, the confusion matrix between the real and predicted classification of each voxel, the mean decrease accuracy of each predictor (grey for non-significant predictor and red for significant predictor), the probability to observe a change of functional connectivity according to the ReHo, fALFF, R^2^, Euclidean distance and number of tracts ranking.

Regarding fALFF and ReHo metrics, our results highlighted a more time-diffused effect (See Fig. 4, Fig. 5, panel A for the localization of the significant clusters, panel B for their size over time and panel C for the effect size over time). In detail, we identified: (i) an increased fALFF in the right putamen (k = 39, significant from 41 to 52 min), the right pons (k = 30, significant from 49 to 69 min) the right inferior temporal cortex (k = 27, significant from 46 to 52 min); (ii) a decreased fALFF in the left posterior medial cortex (k = 44, significant from 67 to 69 min), the left inferior parietal lobule (k = 40, significant from 60 to 68 min; k = 34, significant from 57 to 100 min), the left anterior cingulate cortex (k = 26, significant from 55 to 62 min) and the left superior parietal lobule (k = 25, significant from 61 to 69 min; see Table 2); (iii) an increased ReHo in the right inferior temporal cortex (k = 75, significant from 60 to 71 min), the right thalamus (k = 43, significant from 64 to 70 min), the right pons (k = 36, significant from 70 to 86 min), the right temporal pole (k = 32, significant from 77 to 88 min) and the right lateral orbitofrontal cortex (k = 26, significant from 65 to 100 min); (iv) a decreased ReHo in the posterior medial cortex (k = 1396, significant from 41 to 55 min), the left hippocampus (k = 471, significant from 44 to 56 min), the left inferior parietal lobule (k = 98, significant from 47 to 70 min), the left premotor cortex (k = 45, significant from 61 to 74 min), the right occipital V4 cortex (k = 42, significant from 50 to 63 min), the left primary motor cortex (k = 34, significant from 60 to 67 min), the right caudate nucleus (k = 28, significant from 44 to 47 min) and the right occipital V1 cortex (k = 26, significant from 45 to 61 min; see Table 3).

**Table 2:** Details of the significant clusters for the effects of TUS on fALFF.

Clusters	Peak	Peak [x y z]	T	K	Details	Effect duration after TUS [min]
**1**	Putamen (R)	15.75 17 17.25	4.754	39	Putamen (k = 20) Claustrum (k = 9) S2 (k = 8) Insula (k = 2)	41–52
**2**	Pons (R)	2.5 6–1.25	5.842	30	Pontine fibers (k16) Pontine nucleus (k = 13) Intermediate medulla (k = 1)	49–69
**3**	ITC (R)	18.25 14.5 9.75	4.968	27	Insula (k = 8) Belt area of auditory cortex (k = 6) Inferior temporal cortex (k = 5) Para-insula (k = 2) Claustrum (k = 4) Superior temporal cortex (k = 1)	46–52
**4**	PMC (L)	−2.5 −6 28.5	−4.882	44	Posterior cingulate cortex (k = 19) Posterior medial cortex (k = 17) Superior parietal lobule (k = 6) Ventromedial intraparietal sulcus (k = 2)	67–69
**5**	IPL (L)	−8.5 −11 29.75	−5.242	40	Inferior parietal lobule (k = 23) V2 (k = 11) V4 (k = 5) Superior parietal lobule (k = 1)	60–68
**6**	IPL (L)	−18.25 −6 30.75	−5.383	34	Inferior parietal lobule (k = 34)	57–100
**7**	ACC (L)	−6 13.25 28.5	−5.094	26	Anterior cingulate cortex (k = 18) Primary motor cortex (k = 4) Posterior cingulate cortex (k = 4)	55–62
**8**	SPL (L)	−9.75 1.25 26	−4.628	25	Superior parietal lobule (k = 20) Posterior cingulate cortex (k = 5)	61–69

**Table 3:** Details of the significant clusters for the effects of TUS on ReHo.

Clusters	Peak	Peak [x y z]	T	K	Details	Effect duration after TUS [min]
**1**	ITC (R)	18.25 15.75 0	5.678	75	Inferior temporal cortex (k = 31) Hippocampal formation (k = 27) Medial temporal pole (k = 17)	60–71
**2**	Thalamus (R)	9.75 6 12.25	5.948	43	Geniculate thalamus (k = 23) Ventral thalamus (k = 10) Posterior thalamus (k = 2) Hippocampal formation (k = 2) Zona incerta (k = 2) Reticular thalamus (k = 1) Medial thalamus (k = 1) Mesencephalic reticular formation (k = 1) Substantia nigra (k = 1)	64–70
**3**	Pons (R)	2.5 8.5 0	6.512	36	Pontine fibers (k = 25) Pontine nucleus (k = 11)	70–86
**4**	Temporal pole (R)	14.5 24.25 7.5	5.955	32	Temporal pole (k = 32)	77–88
**5**	Lateral OFC (R)	15.75 29 18.5	5.929	26	Lateral orbito-frontal cortex (k = 23) Caudal orbito-frontal cortex (k = 1) Ventral premotor cortex (k = 2)	65–100
**6**	PMC	−1.25 −4.75 28.5	−6.482	1396	Posterior medial cortex (k = 430) Superior parietal lobule (k = 372) Inferior parietal lobule (k = 220) V2 (k = 94) Primary somato-sensory cortex (k = 77) V4 (k = 76) V3 (k = 73) V1 (k = 24) Midcingulate cortex (k = 11) Vermis 4 (k = 4) Vermis 5 (k = 4) Primary motor cortex (k = 3) Inferior temporal cortex (k = 3) Middle temporal area (k = 3) Belt areas of auditory cortex (k = 2)	41–55
**7**	Hippocampus (L)	−12 2.5 11	−6.366	471	Caudate nucleus (k = 143) Ventral thalamus (k = 58) Posterior thalamus (k = 50) Medial thalamus (k = 48) Dorsal midbrain (k = 26) Hippocampal formation (k = 26) Putamen (k = 24) Lateral midbrain (k = 22) Reticular thalamus (k = 13) Midline thalamus (k = 11) Geniculate thalamus (k = 11) Nucleus accumbens (k = 9) Epithalamus (k = 8) V2 (k = 6) Medial midbrain (k = 4) Diagonal subpallium (k = 3) Pons (k = 2) Primary olfactory cortex (k = 2) Parahippocampal cortex (k = 2) V3 (k = 2) Claustrum (k = 1)	44–56
**8**	IPL (L)	−15.75 7.25 22.25	−5.924	98	Inferior parietal lobule (k = 23) Secondary somato-sensory cortex (k = 23) Insula (k = 18) Putamen (k = 9) Belt areas of auditory cortex (k = 8) Retro-insula (k = 7) Claustrum (k = 6) Core areas of auditory cortex (k = 4)	47–70
**9**	Premotor (L)	−14.5 15.75 32	−5.858	45	Premotor cortex (k = 45)	61–74
**10**	V4 (R)	25.5–3.75 22.25	−5.959	42	V4 (k = 41) V2 (k = 1)	50–63
**11**	M1 (L)	−13.25 9.75 33.25	−4.579	34	Primary motor cortex (k = 29) Premotor cortex (k = 5)	60–67
**12**	Caudate (R)	2.5 24.25 21	−4.095	28	Caudate nucleus (k = 28)	44–47
**13**	V1 (R)	15.75–17 23.5	−6.310	26	V1 (k = 26)	45–61

The random forest models applied on these two metrics highlighted a high general accuracy to detect voxels for which the activity was changed (for fALFF: 85.12%, p < 0.0001; for ReHo: 88.09% p < 0.0001; See Fig. 4, Fig. 5, panel D), with a higher accuracy of true positive for the model on fALFF (89.4% p < 0.0001) than for ReHo (81.7%, p < 0.0001). For fALFF, the metrics with a significant importance were the fALFF (mean decrease accuracy = 2.57% ± 0.09%, p = 0.0009) and the ReHo (mean decrease accuracy = 0.68% ± 0.07,% p = 0.045), for which the model predicted that a fALFF of 0.436 (percentile 9.9) and a ReHo of 0.556 (percentile 2.002) allow having the highest probability of a change induced by TUS (respectively 38.8% and 61% likely). For ReHo, all the metrics were found as significantly important (ReHo: mean decrease accuracy = 5.25% ± 0.04%, p = 0.0009; Euclidean distance: mean decrease accuracy = 4.37% ± 0.04%, p = 0.0009; fALFF: mean decrease accuracy = 2.81% ± 0.04%, p = 0.0009; R^2^: mean decrease accuracy = 0.85% ± 0.04%, p = 0.0009; number of tracts: mean decrease accuracy = 0.36% ± 0.02%, p = 0.0049). After controlling for each metric, the best metrics' value to reach a significant effect were: a ReHo of 0.822 (percentile 91.4, TUS effect of 94.6% likely), a Euclidean distance of 27.25 (percentile 38.83, TUS effect of 30.2% likely), a fALFF of 0.46 (percentile 35.6, TUS effect of 21% likely), a R2 of 0.039 (percentile 59.09, TUS effect of 39.8% likely) and a number of tract of 6 (percentile 96.19, TUS effect of 28% likely).

Regarding the required amount of data to observe these results (see appendix), three sub-groups could be identified for fALFF, as a percentage of the volume for a given region: The right putamen and the right inferior temporal cortex were significantly compromised with reduced data (less than 50% of their volumes with 15/25 data), followed by the left superior parietal lobule, the left inferior parietal lobule, left posterior medial cortex and the right pons (between 50 and 75% of their volumes even if the data amount is decreased to 10/25), while the left anterior cingulate cortex is still close to its initial volume even with only 10/25 of the data (85%). The ReHo metric showed the most robust data starved results (>75% of the initial volumes were maintained with 10/25 data), with correspondent effects in the right caudate nucleus, the right temporal pole, the posterior medial cortex and the left hippocampus (>75% with 10/25 data), followed by the left primary motor cortex and the left inferior parietal lobule (75-50% with 10/25 data), then by the right occipital V4 and V1 cortex, the right thalamus and the right inferior temporal cortex (50-25% with 10/25 data), and finally by the right lateral orbitofrontal cortex and the right pons (25-0% with 10/25 data). Three ReHo clusters were found as significantly related to the potential confound of fMRI acquisition session order (i.e., the right lateral orbitofrontal cortex, the left hippocampus and the left primary motor cortex), while two ReHo clusters were found as significantly related to the number of session (i.e., right thalamus and the left premotor cortex), but none of these survived after Bonferroni correction (see Table 4).

**Table 4:** Effects of session order on the peak of each cluster found as significantly change after TUS.

Metric	Side	location	Group effect	Session effect (all)	Session effect (TUS)
R^2^	Increase	1 (Cereb 5 – L)	**<0.0001**	0.5917	0.1032
		2 (Cereb 5 – L)	**<0.0001**	0.6185	*0.0071*
		3 (V2 – R)	**0.0001**	0.5101	*0.0225*
		4 (OFC – R)	**0.0001**	0.6886	0.0844
		5 (V2 – R)	**0.0001**	0.2967	*0.0049*
		6 (Premotor – R)	**<0.0001**	0.2650	*0.0140*
fALFF	Increase	1 (Putamen – R)	**0.0002**	0.4009	0.2580
		2 (Pons – R)	**<0.0001**	0.1226	0.1249
		3 (ITC – R)	**0.0001**	0.5873	0.6679
	Decrease	4 (PMC – L)	**0.0002**	0.3654	0.2319
		5 (IPL – L)	**<0.0001**	0.2823	0.5797
		6 (IPL – L)	**<0.0001**	0.5581	0.2584
		7 (ACC – L)	**<0.0001**	0.1675	0.7713
		8 (SPL – L)	**0.0003**	0.9797	0.9130
ReHo	Increase	1 (ITC – R)	**0.0001**	0.2442	0.9483
		2 (Thalamus – R)	**<0.0001**	*0.0067*	0.7398
		3 (Pons – R)	**<0.0001**	0.5296	0.1803
		4 (Temporal pole – R)	**0.0001**	0.8029	0.8712
		5 (Lateral OFC – R)	**0.0003**	0.2426	*0.0050*
	Decrease	6 (PMC)	**<0.0001**	0.8922	0.1200
		7 (Hippocampus – L)	**<0.0001**	0.8166	*0.0499*
		8 (IPL – L)	**<0.0001**	0.1173	0.3442
		9 (Premotor – L)	**<0.0001**	*0.0037*	0.4200
		10 (V4 – R)	**<0.0001**	0.9961	0.5157
		11 (M1 – L)	**<0.0001**	0.9505	*0.0141*
		12 (Caudate – R)	**0.0017**	0.1413	0.0875
		13 (V1 – R)	**<0.0001**	0.5869	0.0691

P-values ≤0.05 are highlighted in grey cells while those still significant after Bonferroni correction are indicated in bold.

## 3 Discussion

TUS stimulation of the left hippocampus, as a model highly interconnected brain region (Schultz et al., 2014; Wagner et al., 2016; Small et al., 2011), revealed a twofold temporal pattern in interconnected brain networks. We observed gradual broad increases in functional connectivity during a narrow temporal window, whereas metrics such as fractional amplitude of low-frequency fluctuations (fALFF; which could be interpreted as spontaneous neural activity with the caveat that fMRI BOLD does not directly measure neurophysiological responses (Cordes et al., 2001; Zou et al., 2008)) and regional homogeneity (ReHo; which could be interpreted as local synchronization (Jiang and Zuo, 2016)) revealed both increments and decrements. These effects were observed to typically manifest over a more extended timeframe, rejecting our initial hypothesis of systematic gradually decreasing TUS effects on the neural network. These effects of TUS are not confined solely to the region being stimulated but extend to relatively distant brain regions interconnected through functional pathways associated with the anterior hippocampus. The power of this case is the substantial within-individual power not possible to obtain to date in other animals, the surprising temporal dynamics, diffusivity and consistency of effects revealed, which now allow generating hypotheses for testing in the development of long-lasting TUS effects in humans reliant on fewer scanning session but more individuals. We also report reduced data analyses which also now help to identify how similar results could be obtained in other individuals with reduced power.

### 3.1 TUS has a twofold temporal pattern

Our study unveiled a twofold temporal pattern of brain responses to TUS. On one hand, changes in R^2^ with the used ‘offline’ TUS parameters are primarily confined to a specific time window of 40–60 min post-TUS. This aligns with prior research documenting changes in brain connectivity within intervals ranging from 30 (Fouragnan et al., 2019; Sanguinetti et al., 2020) to 60 min (Folloni et al., 2019; Verhagen et al., 2019). Conversely, modifications in fALFF and ReHo metrics exhibited even longer-lasting effects that, in some cases, extend beyond our measurable timeframe (at least 100 min). This extended duration holds promise for inducing lasting changes in brain function. This finding resonates with existing studies: for example, TUS applied to the sensorimotor network in healthy individuals has been shown to enhance the network’s activity for up to a week after stimulation (Matt et al., 2022). Interestingly, the two measures for which the TUS effects were longer (fALFF and ReHo) correspond to measures of spontaneous brain activity. If they are neurobiologically related, however, they also reveal different brain function metrics and characteristics. Taken together, we highlighted common alterations following TUS (i.e., in the right posterior medial cortex, the left inferior temporal cortex, inferior parietal lobule), while some effects are metric specifics. Thus, while the observed temporally constrained TUS effects are predominantly of medium-term duration (approximately 1 h), some effects can reasonably be assumed to have even longer-term impact, suggesting a possible long-term potentiation (LTP) plasticity. If the mechanisms of TUS on brain plasticity are not known, it was suggested that theta-burst TUS protocols can induce LTP plasticity by modulating astrocytes' activity, themselves inducing NMDA glutamatergic receptors' plasticity (Blackmore et al., 2023; Caffaratti et al., 2024). Our data indicate these mechanisms may be realized differently and for different lengths of time in various brain regions. Our results provide the foundation to study the neural mechanisms for these effects and their longevity in future studies. In addition, our results advance analytical measures that can be used to capture these effects going beyond traditional seed-based measures of network interconnectivity.

The TUS effects were stronger than results from accounting for the temporal order of the fMRI acquisition sessions (across TUS and no-TUS control sessions) and the TUS effects did not appear to carry over to the next sessions. We also show supplemental results, demonstrating how much data might be needed to obtain a fraction of the effects with the fully powered datasets, to guide future studies with less-powered datasets. On the one hand, we found that the R^2^ metric was the measure most reduced when the sample size reduced, but this is also the metric which seems the more related to an accumulated effect over-time (four out of six clusters showed uncorrected significant effects with the fMRI order of acquisition). We could here hypothesize that R^2^ needs several more TUS sessions than the fALFF and ReHo measures before showing significant effects.

Importantly, our study investigated the effect of TUS on awake primate model resting-state connectivity avoiding anesthetic influences in brain-wide network interconnectivity (e.g., changes in excitatory-inhibitory neuronal interactions). This approach better captures the resting-state fMRI network interactions often reliant on resting and awake functional neuroimaging (Hori et al., 2020a; Banks et al., 2020; Li et al., 2023).

### 3.2 TUS effect propagation

Our findings highlight the capacity of TUS-induced effects to extend beyond the initially targeted brain region. Several of them are of importance and could reflect different consequences of applying TUS on the hippocampus.

We observed altered ReHo in both hippocampi (even though only the left received TUS), commencing with decreased ReHo in the targeted hippocampus (44–56 min post-TUS) followed by increased ReHo in the contralateral hippocampus (60–71 min post-TUS). This suggests a compensatory reaction of the contralateral hippocampus to the initial change, a phenomenon previously noted in human patients with unilateral hippocampal dysfunction contributing to epilepsy (Vanicek et al., 2016; Yoo et al., 2019) or related to brain cancer (Liu et al., 2021).

Our results are also perfectly in line with the posterior medial – anterior temporal (PMAT) framework (Ritchey et al., 2015). The posterior parietal cortex was shown to be an effective choice of target to induce indirect neuromodulation in the hippocampus when limited by the shallow depth of stimulation offered by TMS (Freedberg et al., 2019; Hermiller et al., 2018; Thakral et al., 2020; Velioglu et al., 2021; Wang et al., 2014). In our case, the inverse result was observed since hippocampus stimulation altered the posterior medial system (posterior cingulate/precuneus, and inferior parietal lobule). This result could be of importance since this network is related to memory performance in normal ageing (Huo et al., 2018).

Furthermore, we found a pattern of functional changes propagating through the disrupted hippocampus. Many of these changes manifest in the sensorimotor and default mode networks, which share significant connectivity with the hippocampus (Ezama et al., 2021; Hori et al., 2020b; Rosene and Van Hoesen, 1977; Blatt and Rosene, 1998), particularly its anterior portion involved in sensorimotor integration (Small et al., 2011; Bast and Feldon, 2003). Enhanced connectivity between default mode and sensorimotor networks has been linked to post-stroke recovery (Wu et al., 2020), and cross-network connectivity changes are common in psychiatric disorders (Sha et al., 2019).

Also, while R^2^ as a metric predominantly shows an increase within the sensorimotor and default mode networks, fALFF and ReHo exhibit a distinctively different pattern—increasing in subcortical regions (thalamus and pons) and decreasing in cortical areas (posterior medial cortex, inferior and superior parietal lobules, premotor cortex, primary motor cortex, and anterior cingulate cortex). The caudate nucleus, however, displays a decrease in ReHo. This interaction may reflect the pivotal role of basal ganglia in modulating the function of both default mode and sensorimotor networks after TUS neural network perturbation. The broader significance of this phenomenon emerges from observations that disruptions in basal ganglia or pons correlate with decreased functioning in these networks (Chen et al., 2019).

Last, we identified increased R^2^ between the hippocampus and the cerebellum. While the relationship between these two areas was a long-lasting debate, it is now accepted that they could form the “hippobellum” circuit (Froula et al., 2023). This network was especially observed as active during spatial exploration tasks, including in humans (Onuki et al., 2015).

### 3.3 TUS effects could be predicted at an intra-individual scale

We extended our analysis by employing random forest models to determine if brain features collected before TUS could contribute towards explaining and predicting the outcomes we observed. While these models exhibit high accuracy (implying a considerable predictability of TUS outcomes), differences were found regarding the relevant metrics. Specifically, changes in R^2^ are more likely to manifest in brain regions functionally disconnected (i.e., low R^2^) from the target site with relatively high fALFF. Alterations in fALFF were found in brain regions characterized by low fALFF and minimal ReHo. ReHo changes are contingent upon several variables, including very high ReHo, relatively low fALFF, intermediate functional connectivity with the target (i.e., R^2^), moderate distance from the target, and direct anatomical connectivity with the target.

In other words, TUS effects followed a specific pattern which could be anticipated using control no-TUS brain features. This approach parallels earlier applications (Segato et al., 2020), such as adaptative deep brain stimulation in Parkinson’s disease (Krauss et al., 2021), predicting treatment response in potential schizophrenic patients based on rs-fMRI data (Paul et al., 2022), forecasting mood and cognitive changes after transcranial direct current stimulation in major depression patients using rs-EEG (Al-Kaysi et al., 2017), and estimating Parkinson’s disease severity using rs-fMRI ALFF metric (Tian et al., 2020). Subsequent studies will need to validate this predicted pattern using a very high amount of data combined with learning algorithms. If successful, it could pave the way for more individualized TUS long-lasting applications in humans.

### 3.4 Limitations of the study

As with any case study, it is important to recognize that these findings derive from a single primate subject. It has taken years to train this individual for the required scanning stability and to obtain these large amounts of data with and without TUS. This will be extremely difficult to achieve in other primates at least in the immediate future, and most primate neuroscientific studies of this sort conduct a set of case studies with 2–3 animals, often with fewer scanning sessions. Given the robustness of the datasets and the high potential value of this case report being reported to guide future initiatives, the results of this timely case are being reported as a case study with the limitations that need to be considered. Additionally, we conducted data reduction analyses to better establish how much data might be minimally needed for obtaining the reported effects with other individuals. While this limitation inherent to case studies could pose challenges in terms of generalization of the specific results to other individuals, it does confer a distinct advantage in terms of result reliability in this initial study of TUS temporal dynamics in a primate case. This is because the capacity to observe changes within the same individual inherently holds greater robustness than tracking variations across distinct subjects. This enhanced uniformity could also plausibly account for the robust accuracy demonstrated by the random forest models, thereby strengthening the intra-subject consistency of this analytical approach.

Moreover, our findings hinge on the analysis of 52 resting-state fMRI datasets TUS. This sizeable sample contributes to a heightened level of statistical confidence in the reported outcomes. Also, it is pivotal to note that our models are constructed around a TUS effect projected within the timeframe of 0–100 min, whereas the actual data acquisition transpired between the 32nd and 91st minute post-TUS. This discrepancy introduces a limitation with regard to the duration of effect estimated, as the shapes of the curves predicted by our models potentially underestimate the temporal extent. While our estimations remain reliable concerning the ultimate effect, because it took time to prepare the individual for scanning after TUS or sham stimulation, we could not evaluate very short-term effects occurring prior to the 30-min mark post-TUS.

## 4 Conclusion

In conclusion, our investigation in a high-powered awake non-human primate dataset has effectively demonstrated the differential time course of dynamic effects on the primate brain as a neural network. Notably, we have ascertained that while the majority of changes manifest within the initial hour, certain effects might endure over longer periods (>100 min), challenging the assumption that TUS effects gradually decrease over time and are similar in terms of impact across brain neural features. The impact of this stimulation predominantly affected function in the TUS region itself, alongside the default mode and sensorimotor networks. Given the wide-ranging involvement of these networks in diverse cognitive processes and psychiatric conditions, the implications of our findings hold significance for their potential translation to human applications of longer lasting TUS in the future. In addition, our results underscore the feasibility to consider the time dynamic of TUS effects and the openly shared data can be modeled with a reduced dataset to simulate findings with more reasonable amounts of data than can be acquired in humans. This approach could be translated to human datasets with several acquisitions per subjects (informed by our reduced data analyses) or with sequences long enough to capture TUS dynamics.

## 5 Materials and methods

### 5.1 Ethics

All procedures were approved by the Animal Welfare and Ethical Review Body at Newcastle University and by the United Kingdom Home Office (PPL 60/4095, 60/4037 and 70/7976). Experiments complied with the Animal Scientific Procedures Act (1986), the European Directive on the protection of animals used for scientific purposes (2010/63/EU), the United States National Institutes of Health Guidelines for the Care and Use of Animals for Experimental Procedures and were performed with great care and regulatory oversight to ensure the well-being of the animal in the regulated procedures.

### 5.2 Experimental model and study participant details

One Rhesus macaque (*Macaca mulatta*, male, 15 years old, 13.5 kg) was involved in this study which consisted of 21 daily scanning sessions. Among these daily scanning sessions, 12 served as controls with no application of transcranial focused ultrasound stimulation (TUS), while 9 sessions involved TUS targeting the left anterior segment of the hippocampus. Following each session, the acquisition of 1–3 awake T1-weighted and resting-state functional magnetic resonance imaging (rs-fMRI) scans was carried out.

For the TUS condition, MRI scans (∼11 min duration each) were acquired between 32 and 91 min following TUS, allowing for the time needed to prepare the animal for scanning after TUS. Two rs-fMRI were removed due to data acquisition quality issues during the acquisitions (one for each condition). In total, 52 rs-fMRI datasets were acquired, 27 for the control condition and 25 for the TUS condition. In addition, 5 Diffusion-weighted images (DWI) were acquired during the control state.

### 5.3 Ultrasound stimulation

Our TUS protocol emulated a commonly used primate “*offline*” TUS protocol (Folloni et al., 2021). The single-element H115-MR transducer (manufacturer: Sonic Concepts Inc., Bothell, USA; number of elements: 1, radius of curvature: 64 mm) was used with a coupling cone filled with degassed water and sealed with a latex membrane to reach a focal depth of 51.74 mm. The following parameters were used for the stimulation, controlled through a digital function generator (KeySight 33500B Trueform, Santa Rosa, CA, USA): fundamental frequency: 250 kHz, pulse duration: 30ms, pulse repetition interval: 100ms, pulse repetition frequency: 10Hz, duty cycle: 30%, I_SPPA_: 15.5W/cm^2^; total duration: 40 s. A 75-Watt amplifier (Krohn-Hite Model 7500, Brockton, MA, USA) was used to deliver the required power to the transducer. An oscilloscope (Tektronix TBS 1032B, Beaverton, OR, USA) was used to monitor the voltage delivered. The recorded peak-to-peak voltage was kept constant throughout the stimulation. Based on simulations achieved using k-plan, sonification outputs were estimated in water (maximum peak pressure: 682 kPa; I_sppa_: 15.5W/cm^2^; I_spta_: 4.26W/cm^2^) and *in situ* (maximum peak pressure: 548 kPa; I_sppa_: 10W/cm^2^; I_spta_: 2.76W/cm^2^; Mechanical Index: 1.1; Maximal increase temperature: +2.61 °C). The sonicated tissues were considered when the applied pressure was ≥0.2 MPa.

To direct TUS to the target region, each individual animal’s structural MR image was registered to its head with a frameless stereotaxic neuronavigation system (Rogue Research, Montreal, CA). By recording the positions of both the ultrasound transducer and the head with an infrared tracker it was then possible to co-register the ultrasound transducer with respect to the MRI scan to position the transducer over the trajectory leading to the left anterior hippocampus (x = −14.5, y = −7.5, z = −12.5). The ultrasound transducer/coupling cone was placed directly onto previously shaved skin prepared with conductive gel (SignaGel Electrode; Parker Laboratories Inc.) to ensure ultrasonic coupling between the transducer and the animal’s scalp.

### 5.4 Neuroimaging data acquisition

We used a 4.7 T vertical magnet running ParaVision 5.1 (Bruker, BioSpin GmbH, Ettlingen, Germany) and equipped with a 4-channel phase-array coil (https://www.wkscientific.com) for data acquisition. All neuroimaging data were acquired without any anesthesia and with eyes open, with the primate continuously monitored and trained to infrequently receive juice rewards for staying awake but relaxed during the scanning runs. Juice rewards were delivered every few minutes to not substantially interfere with the scanning data acquisition. The neuroimaging data collection was based on three sequence types: 1) DWI for tractography (∼75 min per scanning; 60 diffusion directions; 4 B = 0 images; B = 850 s/mm^2^; 5 repetitions; repetition time [TR] = 14.2sec; echo time [TE] = 58ms; field of view [FOV] = 88 × 88 mm; 56 slices of 1 mm^3^ isotropic voxels; 2x parallel acceleration with GeneRalized Autocalibrating Partial Parallel Acquisition reconstruction (GRAPPA) reconstruction; 1.15 partial Fourier acceleration), 2) echo-planar imaging (EPI) for rs-fMRI (∼11 min per scanning run; 250 vol acquired; TR = 2.6sec; TE = 20ms; FOV = 88 × 88 mm; 48 interleaved slices of 1.2 mm^3^ isotropic voxels; Bandwidth = 150 kHz, 2x acceleration with GRAPPA reconstruction), and 3) 3D-modified driven equilibrium Fourier transform (MDEFT) for anatomical imaging (2D MPRAGE sequence; Inversion time [TI] = 800ms; TE = 3.8ms; echo-TR = 19 ms; TR = 2000 ms; FOV = 132 × 132; 120 slices of 0.8 mm^3^ isotropic voxels).

### 5.5 Neuroimaging data pre-processing and metrics extraction

The pre-processing of the rs-fMRI was achieved with AFNI (Cox, 1996; Cox and Hyde, 1997) by using the standard pre-processing steps (Jung et al., 2021). First, the T1 was non-linearly realigned to match the rs-fMRI. Then, the T1 was pre-processed by removing the skull, segmentizing the different tissues and realigning to the template space (i.e., NMTv2 (Jung et al., 2021; Seidlitz et al., 2018)). The rs-fMRI were pre-processed with a slice timing correction, despike and motion correction performed in the native space as well as a realignment to the mean image. Then, the partially pre-processed rs-fMRI were realigned to the template space by using the conversion matrix obtained during the T1 pre-processing. Lastly, we smoothed (3 mm) and detrended the rs-fMRI using motion as a nuisance variable and band-passed from 0 to 0.12Hz. At this step, 3 different maps were extracted, each representing a rs-fMRI metric: (i) a map of the strength of functional connectivity (R^2^) between all voxels and the targeted left anterior hippocampus (so-called “seed-based connectivity”) by using a sphere (2.5 mm diameter, x = −14.5, y = −7.5, z = −12.5) as seed; (ii) a map of fractional amplitude of low-frequency fluctuation (fALFF, which represents the spontaneous brain activity); (iii) and a map of regional homogeneity (ReHo, with 27 neighbors, which represents the local temporal homogeneity of the regional brain activity; see Fig. 2B).

The pre-processing of the DWI data was achieved using both AFNI and MRtrix3 (Tournier et al., 2019) using a probabilistic reconstruction and in the following order: denoising, Gibbs artifacts removing, FSL pre-processing, B1 field inhomogeneity correction for bias caused by the coil spatial sensitivity, unsupervised estimation of brain tissues multi-shell multi-tissue fibre orientation distributions estimation. Then, 10 million tracts were generated using a seed-based approach (i.e., all tracts which cross to the TUS target; sphere of 2.5 mm diameter, x = −14.5, y = −7.5, z = −12.5) before we corrected them using the SIFT approach (Spherical-deconvolution Informed Filtering of Tractograms (Smith et al., 2013)) and decreased them to 1 million. We then moved the tracts to the template space, allowing us to obtain a seed-based structural connectivity value of every voxel with the TUS target.

### 5.6 Statistical analysis

All the statistical analyses were achieved using R (R Core Team, 2021) employing the same approach for the three considered metrics (R^2^, fALFF, ReHo; see Fig. 2).

First, for each voxel we estimated a beta generalized additive model (GAM) with the metric as a dependent variable and the time after TUS (measured in minutes) as predictor. For the MRI corresponding to the control sessions, a time value of 0 was used to force the model to have an intercept corresponding to a “*normal*” state. For the rs-fMRI corresponding to the TUS sessions, the median time of the acquisition moment were used (e.g., if the rs-fMRI started 30 min after TUS and ended 40 min after TUS, we gave the value of 35). This model allows drawing a smoothed relation between the time and the metrics, for which the estimation is always comprised between 0 and 1. For each minute between 0 and 100 min after TUS, we extracted the prediction of the model and its standard deviation. Then, for each minute, we computed a two-sample *t*-test between the values of the control sessions and the estimated values by the model. We then extracted a brain map with the lowest p-value observed for each voxel. We considered as significant any clusters for which the minimal observed p-value were ≤0.001 and with a cluster size of at least 10 voxels for R^2^, and 25 voxels for fALFF and ReHo.

Second, for all clusters found as significant, we extracted the time course of the difference with the control sessions (t values obtained during the t-tests) for all voxels, the size of the cluster (proportion of voxels significant at each time point), and the variability of the time course (coefficient of variation; see appendix for the results details of each significant clusters).

Third, we aimed to determine if our results could have been predicted by using brain information obtained during the control no-TUS condition. Therefore, we built a data frame with several data for all voxels of grey matter, excluding those of the target (sphere of 2.5 mm diameter, x = −14.5, y = −7.5, z = −12.5). The considered data were the mean (between all MRI acquired during the control condition) of the R^2^, ReHo and fALFF, the Euclidean distance to the seed and the mean number of tracts. We also generated 3 additional columns which corresponded to the information to predict, i.e., if we observed for the voxel, a change of R^2^, ReHo or fALFF (categorized as “1” if a change was observed and as “0” if no we observed no change). We then applied a random forest model associated to a permutation approach (500 trees and 1000 permutations) to classify each voxel as having (1) or not (0) change. The permutations allowed to calculate a null distribution of the importance of each variable in the accuracy of the model and therefore to attribute a p-value to its importance. The importance of each predictor is measure by the “mean decrease accuracy” which corresponds to the loss of accuracy of the full model if the predictor is removed. We considered a mean decreasing accuracy as significant when p-value ≤0.05. Then, we build a data frame with the significant predictors ordered by percentiles to identify the metrics values which are the most likely to be related to a change of brain functioning after TUS.

Fourth, we tried to identify the minimal amount of data required to find our results. We selected all the voxels found as significant and ran the analyses a second time, by selectively removing some data. In detail, we selected one voxel, removing randomly one of the MRI (TUS condition) and ran the GAM model. We repeated this step 100 times by randomly removing another data point. If the voxel was found as below our threshold (p < 0.001) in at least 50% of the simulations, we considered the voxel as still significant. We then ran again the analysis but by removing two data points, then three, until only 10 were still present (the minimum number of data points required to run the GAM model). This approach allows us to determine how many data points are required to found a cluster as still significant. The results could be found in appendix.

Fifth, using regressions analyses, we assessed the relationship between the effects we found and the session order for all the fMRI runs (both control and TUS conditions, which could be interpreted as a stability measure over the study, so mostly as a noise and/or non-specific temporal order effect control) and for the effect on the next TUS session only (addressing the question of repeated TUS effects). The regressions were applied on the coordinates of the peak of each clusters found as significant. Bonferroni correction was applied to correct for multiple comparison.

## CRediT authorship contribution statement

**Cyril Atkinson-Clement:** Methodology, Formal analysis, Writing – original draft, Visualization. **David Howett:** Investigation, Writing – review & editing. **Mohammad Alkhawashki:** Writing – original draft. **James Ross:** Writing – review & editing. **Ben Slater:** Investigation, Writing – review & editing. **Marilyn Gatica:** Writing – review & editing. **Fabien Balezeau:** Methodology, Writing – review & editing. **Chencheng Zhang:** Writing – review & editing. **Jerome Sallet:** Methodology, Writing – review & editing. **Chris Petkov:** Conceptualization, Methodology, Investigation, Writing – review & editing, Funding acquisition, Supervision. **Marcus Kaiser:** Conceptualization, Methodology, Writing – review & editing, Funding acquisition, Supervision.

## Declaration of competing interest

The authors declare the following financial interests/personal relationships which may be considered as potential competing interests: Marcus Kaiser reports financial support was provided by 10.13039/501100000266Engineering and Physical Sciences Research Council. Marcus Kaiser reports financial support was provided by Guangci Professorship Program of Rui Jin Hospital. Chris Petkov reports financial support was provided by 10.13039/501100000265Medical Research Council. If there are other authors, they declare that they have no known competing financial interests or personal relationships that could have appeared to influence the work reported in this paper.

## Data Availability

Data will be made available on request.
