# Supplementary material for: Temporal dynamics of offline transcranial ultrasound stimulation
Source: Curr Res Neurobiol. 2025 Mar 6;8:100148. doi: 10.1016/j.crneur.2025.100148 (PMC11950745; doi:10.1016/j.crneur.2025.100148)
Supplement: Multimedia component 1 [file mmc1.docx]

**Temporal Dynamics of Offline Transcranial Ultrasound Stimulation**

Cyril Atkinson-Clement^1*^, David Howett^2^, Mohammad Alkhawashki^1^, James Ross^1^, Ben Slater^3^, Marilyn Gatica^1,4^, Fabien Balezeau^3^, Chencheng Zhang^5,6^, Jerome Sallet^7,8^, Chris Petkov^3,9 ⴕ^, Marcus Kaiser^1,10,11 ⴕ^

^1^ Precision Imaging, School of Medicine, University of Nottingham, United Kingdom.

^2^ School of Psychological Science, University of Bristol, United Kingdom.

^3^ Biosciences Institute, Newcastle University Medical School, United Kingdom.

^4^ NPLab, Network Science Institute, Northeastern University London, London, United Kingdom.

^5^ Department of Neurosurgery, Ruijin Hospital, Shanghai Jiao Tong University School of Medicine, China.

^6^ Shanghai Research Center for Brain Science and Brain-Inspired Intelligence, China.

^7^ Wellcome Centre for Integrative Neuroimaging, Department of Experimental Psychology, University of Oxford, United Kingdom

^8^ Univ Lyon, Université Lyon 1, Inserm, Stem Cell and Brain Research Institute U1208, Bron, France

^9^ Department of Neurosurgery, University of Iowa, USA.

^10^ School of Computing Science, Newcastle University, United Kingdom.

^11^ Rui Jin Hospital, Shanghai Jiao Tong University, Shanghai, China.

^ⴕ^ These authors contributed equally.

**Appendix**

**
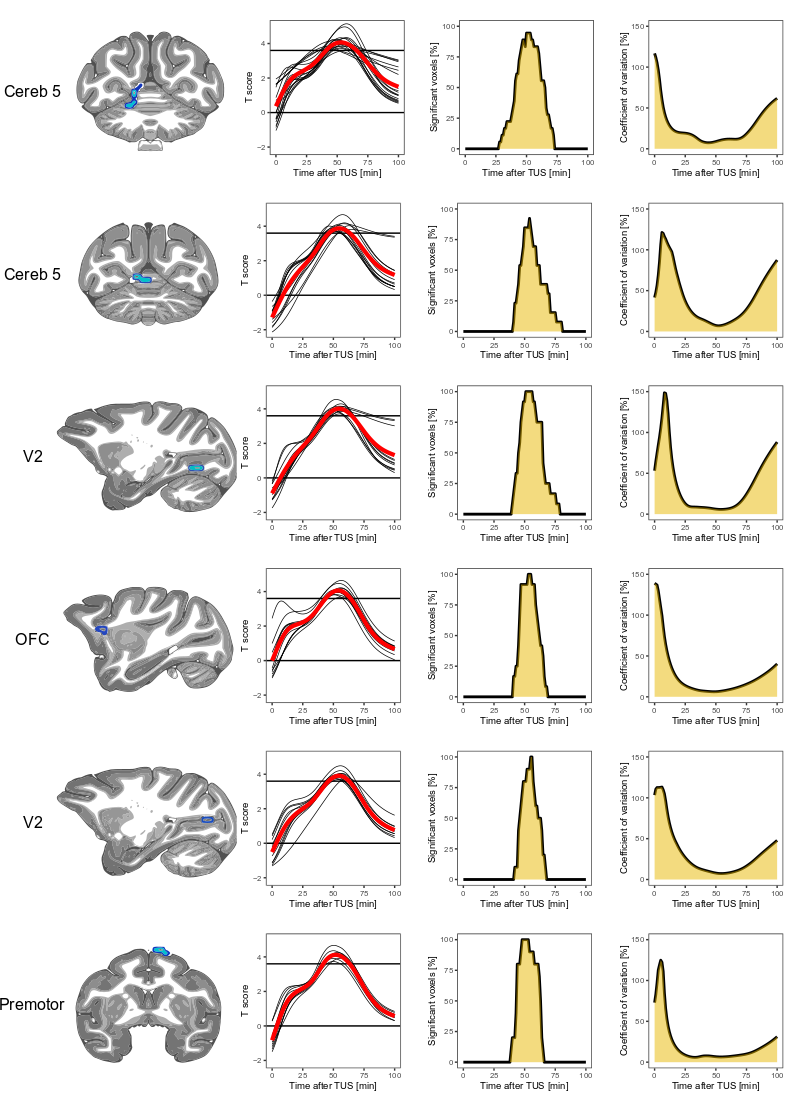
**

**Figure S1. Details of the significant clusters for the effects of TUS on R^2^.**

The first column represents the significant clusters. The second one shows the time course of the T-scores for all voxels of the clusters. The third column corresponds to the percentage of voxels which reach the threshold for significance over-time. The fourth column corresponds to the coefficient of variation of the T-scores of all voxels of the clusters.

**
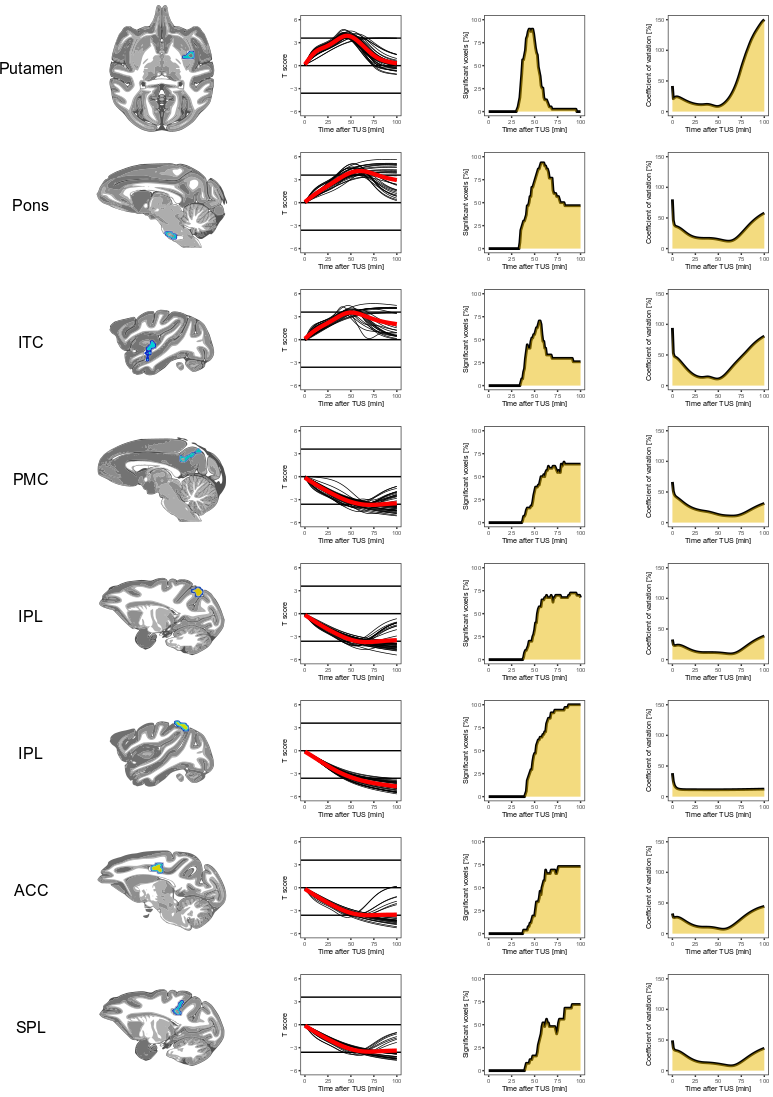
**

**Figure S2. Details of the significant clusters for the effects of TUS on fALFF.**

The first column represents the significant clusters. The second one shows the time course of the T-scores for all voxels of the clusters. The third column corresponds to the percentage of voxels which reach the threshold for significance over-time. The fourth column corresponds to the coefficient of variation of the T-scores of all voxels of the clusters.

**
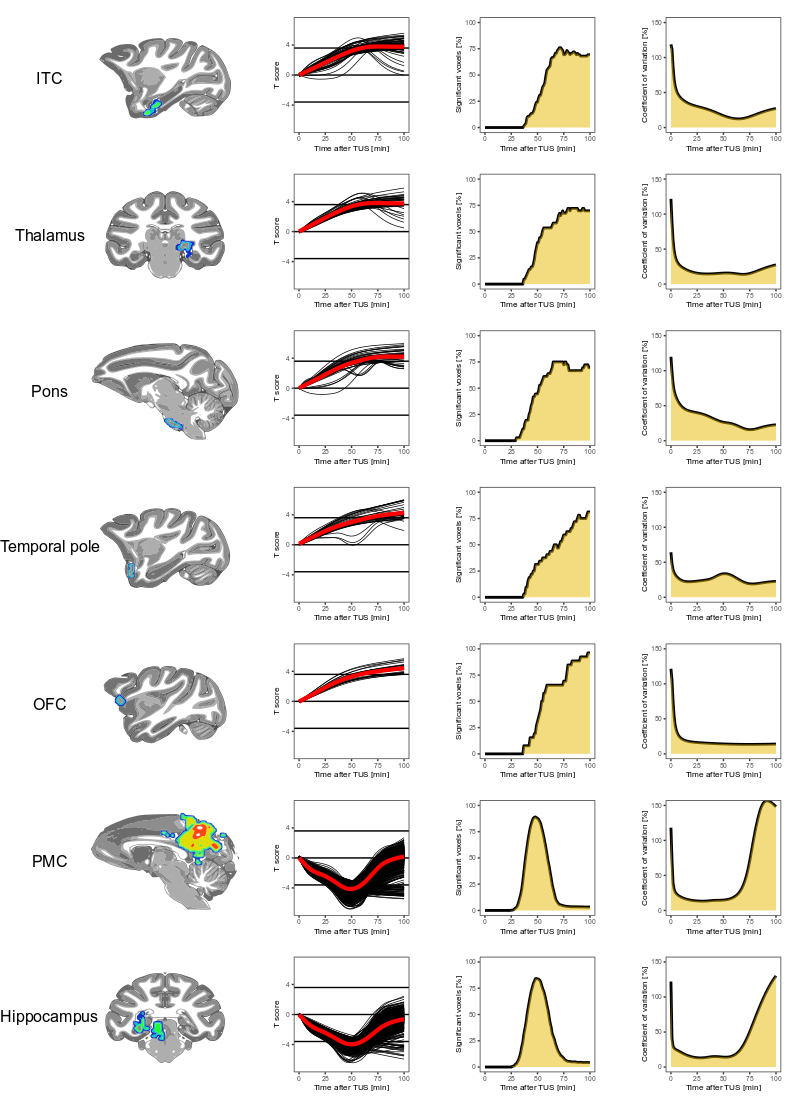
**

**Figure S3 (part 1). Details of the significant clusters for the effects of TUS on ReHo.**

The first column represents the significant clusters. The second one shows the time course of the T-scores for all voxels of the clusters. The third column corresponds to the percentage of voxels which reach the threshold for significance over-time. The fourth column corresponds to the coefficient of variation of the T-scores of all voxels of the clusters.

**
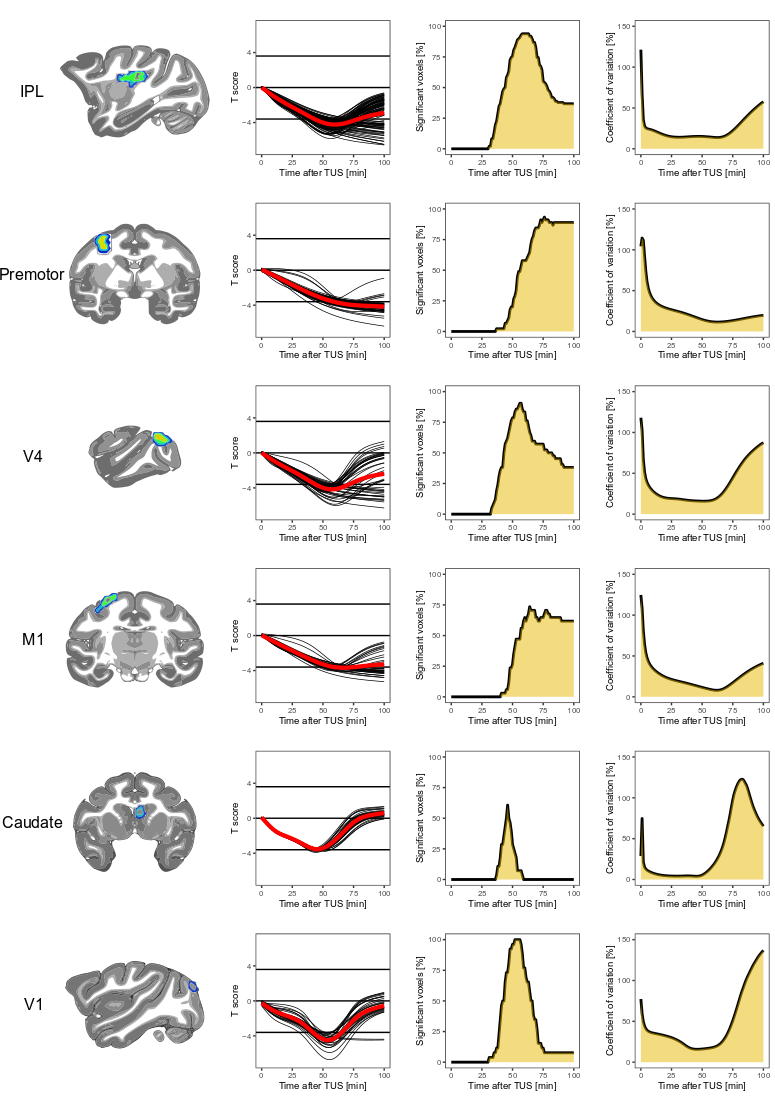
 Figure S3 (part 2). Details of the significant clusters for the effects of TUS on ReHo.**

The first column represents the significant clusters. The second one shows the time course of the T-scores for all voxels of the clusters. The third column corresponds to the percentage of voxels which reach the threshold for significance over-time. The fourth column corresponds to the coefficient of variation of the T-scores of all voxels of the clusters.


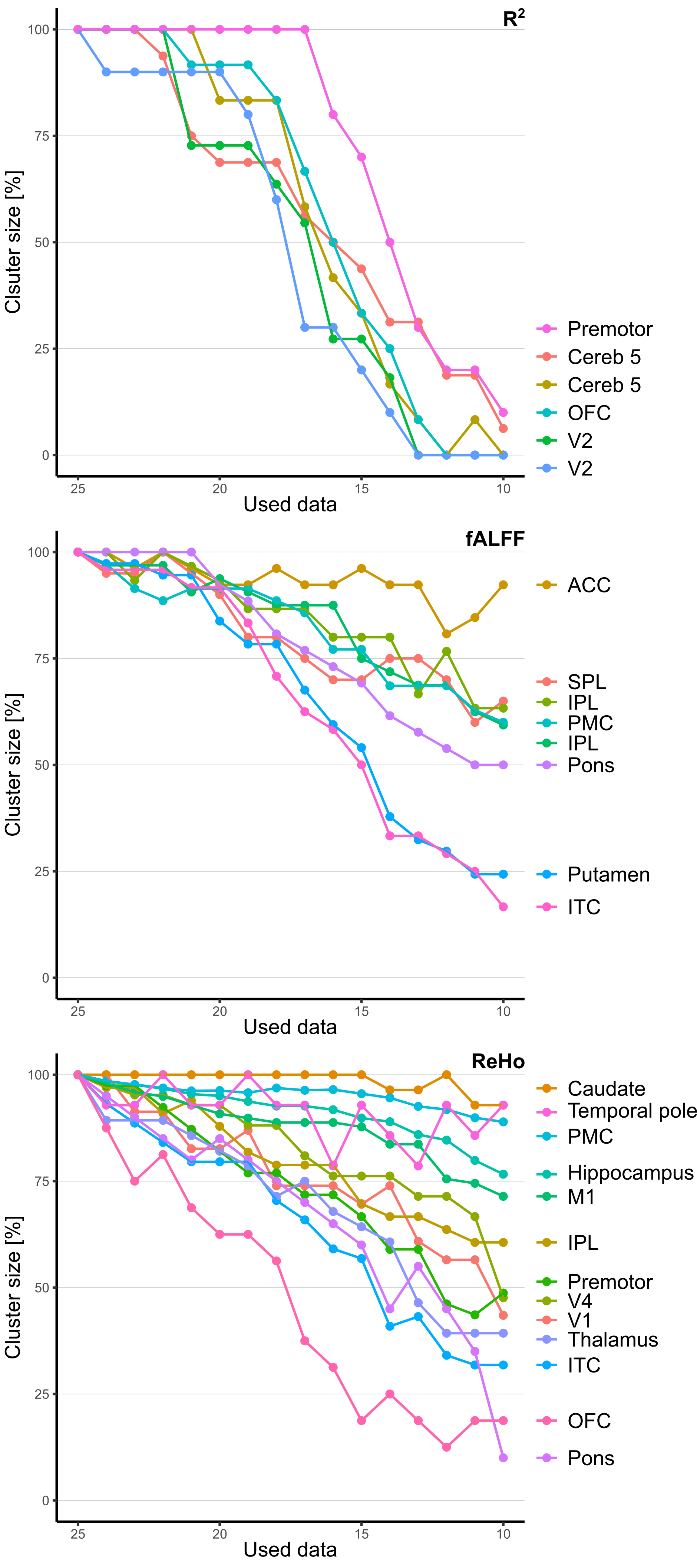


**Figure S4. Minimum amount of data required to observe our results.**

The three graphics (from top to bottom: R2, fALFF and ReHo) represent the percentage of each cluster which is still significant after randomly removing some data points.
